# Trace Level Determination of Saccharides in Pristine Marine Aerosols by Gas Chromatography—Tandem Mass Spectrometry

**DOI:** 10.3390/toxics9040086

**Published:** 2021-04-17

**Authors:** Na-Rae Choi, Yong-Jun Yoon, Ki-Tae Park, Ki-Ae Kim, Yong-Pyo Kim, Yun-Gyong Ahn, Ji-Yi Lee

**Affiliations:** 1 Department of Environmental Science and Engineering, Ewha Womans University, Seoul 03760, Korea; naraechoi9874@ewha.ac.kr (N.-R.C.); rldo428@gmail.com (K.-A.K.); 2 Division of Polar Climate Sciences, Korea Polar Research Institute, Incheon 21990, Korea; yjyoon@kopri.re.kr (Y.-J.Y.); ktpark@kopri.re.kr (K.-T.P.); 3 Graduate Program in System Health Science and Engineering, Department of Chemical Engineering and Materials Science, Ewha Womans University, Seoul 03760, Korea; yong@ewha.ac.kr; 4 Western Seoul Center, Korea Basic Science Institute, Seoul 03759, Korea

**Keywords:** Antarctica, marine aerosol, gas chromatography, tandem mass spectrometry, saccharides, IBRV Araon

## Abstract

The quantification and identification of saccharides in pristine marine aerosols can provide useful information for determining the contributions of anthropogenic and natural sources of the aerosol. However, individual saccharide compounds in pristine marine aerosols that exist in trace amounts are difficult to analyze due to their low concentrations. Thus, in this study, we applied gas chromatography–tandem mass spectrometry (GC-MS/MS) in multiple reaction monitoring (MRM) mode to analyze the particulate matter with an aerodynamic diameter equal or less than 2.5 μm (PM_2.5_) samples, and the results were compared with those of conventional GC-MS. To investigate the chemical properties of pristine marine aerosols, 12 PM_2.5_ samples were collected while aboard Araon, an ice-breaking research vessel (IBRV), as it sailed from Incheon, South Korea to Antarctica. The method detection limits of GC-MS/MS for 10 saccharides were 2–22-fold lower than those of GC-MS. Consequently, the advantages of GC-MS/MS include (1) more distinct peak separations, enabling the accurate identification of the target saccharides and (2) the quantification of all individual saccharide compounds with concentrations outside the quantifiable range of GC-MS. Accordingly, the time resolution for sampling saccharides in pristine marine aerosols can be improved with GC-MS/MS.

## 1. Introduction

Pristine marine aerosols originate from natural sources but are also produced by anthropogenic activities [1]. These aerosols affect the climate directly by scattering and absorbing solar radiation and indirectly by functioning as cloud condensation nuclei (CCN) [1]. The direct effect depends on the chemical composition, size, and optical properties of the aerosols; the indirect effect is based on changes in aerosol concentration levels. Furthermore, the formation and properties of clouds are sensitive to relatively minor changes in aerosol concentrations, particularly in remote and polar areas [2]. Therefore, the chemical compositions of marine aerosols must be understood. 

Organic particulate matter in the atmosphere can originate from primary emission sources as well as atmospheric reactions [1]. Therefore, organic compounds in aerosols have been used as markers to identify the primary emission sources and atmospheric reactions that contribute to particulate matter in the atmosphere [3,4,5]. Among the widely analyzed organic compounds in atmospheric aerosols are saccharides, one of the major classes of water-soluble organic compounds (WSOC), which originate from two principal sources: (1) anthropogenic sources such as coal combustion and biomass burning and (2) natural sources such as microbial detritus, soil biota, plants, and biomass burning. Monosaccharides such as arabinose, ribose, xylose, fructose, mannose, galactose, and glucose are largely emitted from biogenic detritus [6,7,8,9,10]. Disaccharides such as sucrose and maltose are emitted from plant and soil biota [6,7,8,9,10], and anhydro-saccharides, including levoglucosan, mannosan, and galactosan, are produced by biomass burning [4,9,10,11,12]. Therefore, saccharides have been proposed as markers of aerosol sources when anthropogenic and biogenic sources might be intermingled [10].

Previous studies that have analyzed saccharides have been conducted primarily in urban and rural areas [3,13,14,15,16], but some have been carried out in polar environments [10,17], as shown in Table 1. The separation of saccharides in extracted samples of atmospheric aerosols has been performed using liquid chromatography (LC), as applied in the studies by Wan and Yu (2006) [14] and Yttri et al. (2007) [16], as well as gas chromatography (GC), as carried out by Wang et al. (2011) [18], Nolte et al. (2001) [13], and Choi et al. (2016) [3,13,15]. Choi et al. (2016) [3], Nolte et al. (2001) [13], Wan and Yu (2006) [14], Fu et al. (2009) [17], Medeiros and Simoneit (2007) [19], and Wang et al. (2011) [15] adopted quadrupole mass spectrometry (MS) to detect saccharides. Typically, studies using GC-MS to analyze saccharides have performed the derivatization step using bistrifluoroacetamide (BSTFA) and trimethylchlorosilane (TMCS) [3,13,15,17,19]. However, Barboro et al. (2015) [10] investigated the concentrations of saccharides in marine and Antarctic regions by using high-pressure anion exchange chromatography (HPAEC)-MS without a derivatization step.

Instrumental analysis using GC coupled with single quadrupole MS (GC-MS) has been the preferred method to comprehensively characterize the emission sources and atmospheric behaviors of aerosols by simultaneously analyzing saccharides with other organic compounds, such as n-alkanes, polycyclic aromatic hydrocarbons, fatty acids, and dicarboxylic acids. However, in previous studies, the method detection limits (MDLs) that were obtained using GC-MS were not sufficiently low enough for the analysis of saccharides in the Antarctic region, as indicated in Appendix A. The ambient concentrations of saccharides in the Mario Zuchelli Station, Antarctica, ranged from 0.4 to 664.4 ng/m^3^, and the GC-MS MDLs reported in previous studies [3,19] were too high to detect ambient saccharides in pristine environments (Appendix A).

Analyzing saccharides in remote and marine regions, including the Antarctic region, requires a longer sampling time or a more sensitive analytical method. When adopting a longer sampling time, samples can be contaminated, and the temporal resolution of the sampling may not be adequate to identify the main contributors of the saccharides, which might exist in trace concentrations in remote and marine regions. Therefore, adopting a more sensitive analytical technique than GC-MS should be considered for the analysis of saccharides in the particulate matter (PM) samples collected in remote and marine environments. 

This study aimed to evaluate the performance of GC coupled with a tandem mass spectrometer (GC-MS/MS) in analyzing trace amounts of saccharides in PM samples collected in marine and remote regions. The results were compared to those obtained using conventional GC-MS. The MDLs of the target saccharides obtained using GC-MS/MS were lower because the baseline noise was reduced, and thus, the signals of the analytes were increased. Consequently, GC-MS/MS can be used for the accurate identification, quantification, and daily profiling of trace concentrations of the target saccharides in PM samples collected in pristine marine environments, including the Antarctic region.

## 2. Materials and Methods

### 2.1. Chemicals and Reagents

Standard solutions of 10 saccharides (arabinose, ribose, levoglucosan, xylose, fructose, mannose, galactose, glucose, sucrose, and maltose; see Table 2 for their chemical information) for GC-MS and GC-MS/MS were purchased at concentrations of 1000 μg/mL in dichloromethane from Sigma Aldrich (MERCK KGaA, Darmstadt, Germany). The deuterium-labeled internal standard levoglucosan-C_13_ was purchased from C/D/N Isotopes (Quebec City, QC, Canada) and used for quality assurance/quality control (QA/QC). 

A standard working solution (1–1000 μg/mL) was prepared and then stored at −20 °C before use. GC analytical-grade organic solvents (methanol and dichloromethane) were purchased from Burdick & Jackson (Charlotte, NC, USA). 

### 2.2. PM_2.5_ Sampling 

**Figure 1:**
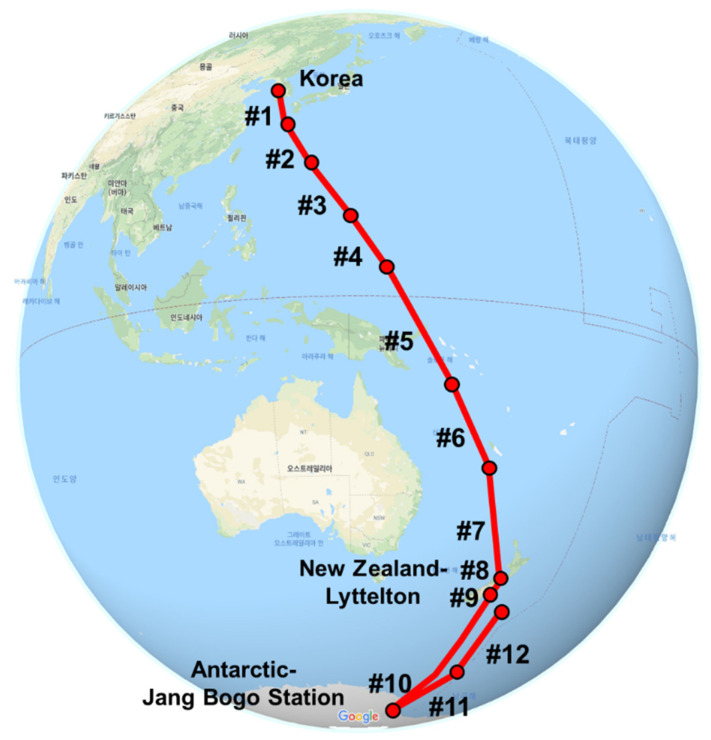
Sampling route taken by the Ice Breaking Research Vessel (IBRV) Araon from Incheon to the Antarctic Jang Bogo Station for the period of 2018.10.31 to 2018.12.14 (Map data: © 2020 Google, ORION-ME).

PM_2.5_ samples were collected while aboard Araon, an Ice-Breaking Research Vessel (IBRV), which sailed from Incheon, South Korea, on 31 October 2018, stopped at Lyttelton, New Zealand, and reached the Antarctic Jang Bogo Station on 7 December 2018 (Figure 1). The sampling concluded in the South Pacific Ocean on 11 December 2018 (Figure 1). PM_2.5_ samples were collected using a high-volume air sampler installed on the third deck facing the bow, which was located on the front deck of the ship and far away from the ship exhaust. The sampler was operated with a wind sector controller. One possible contamination source was a kitchen vent (first deck) with a flexible exhaust line that was vented behind the sampling location. The sampling information is detailed in Table 3. 

A total of 12 samples were collected on 203 × 254 mm quartz microfiber filters (Whatman, 1841–865). Before sampling, the filters were heated at 450 °C for 24 h to remove absorbed organics. After collecting the samples, the filters were wrapped in aluminum foil and stored in a freezer at −20 °C until analysis to minimize contamination. Because sample #10 was damaged during sampling, subsequent analytical procedures were performed without the filters used to collect this sample. The sampling volume of #1 could not be measured because conditions were too unsteady for the wind sector controller, so the saccharides in sample #1 were not quantified. 

### 2.3. Analytical Procedure

Half of the filters were extracted using ultrasonic agitation twice for 30 min with a mixture of dichloromethane and methanol (3:1, *v*/*v*). Before extraction, the isotope-labeled internal standard (levoglucosan-C_13_) was used to spike the samples. All extracts were reduced to 100 mL using a concentrator (Zymark Turbo Vap 500) under a pure nitrogen stream at 40 °C. The extracted samples were cleaned using a syringe filter (Teflon filter, ID 25 mm, pore size 0.45 μm). The extract was reduced further by a gentle solvent evaporator with a stream of high-purity nitrogen to a final volume of 0.5 ± 0.1 mL. 

For the derivatization of sugar compounds, the elution solvent was evaporated completely. After that, BSTFA containing 1% TMCS (50 μL) and pyridine (100 μL) was added and heated at 75 °C for 1 h (REACTI-THERM #18822 Heating module, Thermo Scientific). The derivatized samples were divided into two vials for analysis using GC-MS and GC-MS/MS. 

GC-MS analysis was conducted on a Hewlett Packard 7890A GC equipped with a 5975 mass-selective detector (Agilent Technologies, Santa Clara, CA, USA) in selected ion monitoring (SIM) mode. GC-MS/MS analysis was performed on an Agilent 7890B GC equipped with a 7010 mass-selective detector triple quadrupole MS system in multiple reaction monitoring (MRM) mode. 

A 1 μL sample was injected in the splitless mode at 240 °C. Chromatographic separation was achieved using a DB-5MS (30 m × 0.25 mm I.D; 0.25 mm film thickness; fused with 5% diphenyl and 95% dimethylpolysiloxan) column. The GC oven temperature was maintained at 60 °C for 1 min, ramped to 160 °C at a rate of 20 °C per minute, and then maintained for 1 min. The GC oven temperature was ramped to 210 °C at a rate of 4 °C per minute, maintained for 1 min, ramped again to 260 °C at a rate of 20 °C per minute, and then maintained for 1 min. Finally, the GC oven temperature reached 310 °C at a rate of 4 °C per minute and was maintained for 5 min. 

Single MS was operated in electron impact (EI) mode at 70 eV, and scanning was performed from 40 Da to 550 Da at a source temperature of 230 °C. The acquisition was conducted in SIM mode. The transfer line and ion source temperatures were 245 °C and 300 °C, respectively. The MS was tuned in EI mode at 70 eV in MRM mode. The conditions for SIM mode (MS analysis) and MRM mode (MS/MS analysis) are reported in Table 4. 

A QA/QC experiment was carried out to measure MDLs, recovery, and linearity using spiked analytical samples following the same analytical procedures stated above. The MDLs were measured using GC-MS/MS in MRM mode (1.38–11.70 pg/μL) at a level of 30 pg/m^3^ (*n* = 10) and GC-MS in SIM mode (5.57–31.37 pg/μL) at a level of 6 pg/m^3^ (*n* = 10), as reported in Table 5. As shown in Table 5, the MDLs of GC-MS/MS were generally lower than those of GC-MS. The recoveries of target saccharides were measured using GC-MS in SIM mode (from 40% (mannose) to 109% (levoglucosan); average: 79%), and linearity was measured using both GC-MS/MS in MRM mode (R^2^ > 0.99) and GC-MS in SIM mode (R^2^ > 0.99); the linear ranges of GC-MS/MS and GC-MS are shown in Table 5. The linearities of GC-MS/MS and GC-MS were sufficiently reliable to quantify the target saccharides, and the lowest values in the linear range of GC-MS/MS were lower than those of GC-MS. 

Organic carbon (OC) and elemental carbon (EC) were measured with an OC/EC analyzer (Model 5L, Sunset Laboratory Inc., Tigard, OR, USA) simultaneously with saccharides in the same filter (10.5 cm^2^). The OC and EC concentrations of each sample are listed in Table 6. The analysis protocol followed the National Institute of Occupational Safety and Health (NIOSH) Method 5040 based on thermal-optical transmittance (TOT) [20]. The average precision of the OC analysis with an authentic sucrose standard was 93 ± 2%, with a relative standard deviation (RSD) of 8 ± 4%. Details on the OC and EC analysis are presented elsewhere [21]. 

## 3. Results and Discussion

### 3.1. Improvement of Peak Separations of Saccharides Using GC-MS/MS

**Figure 2:**
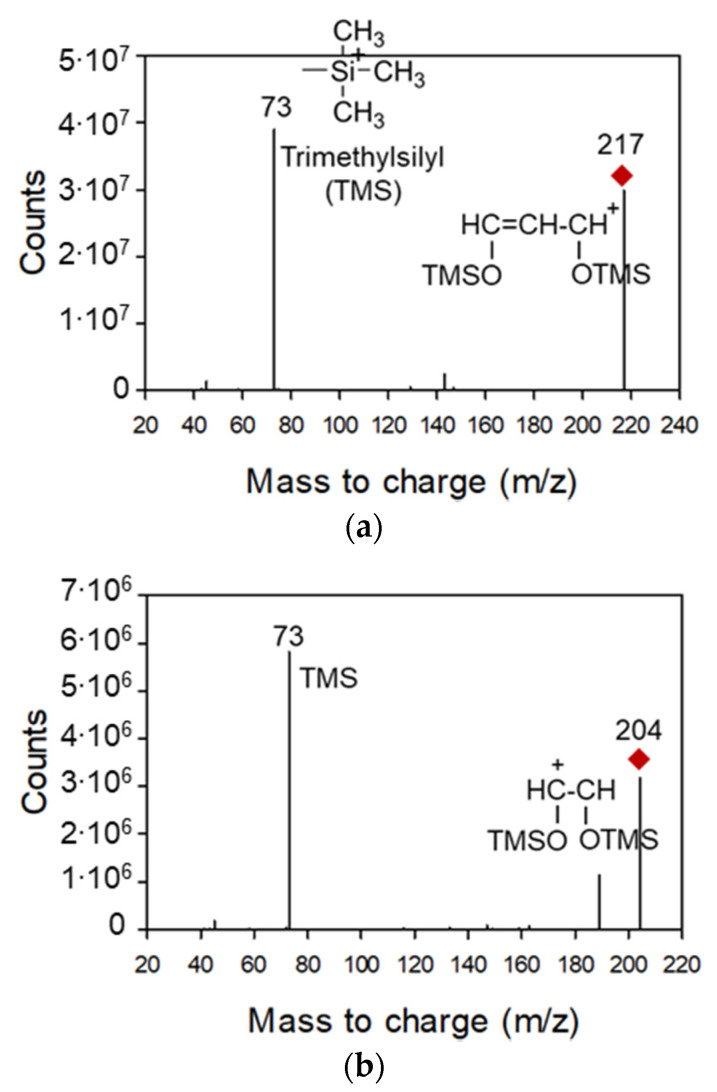

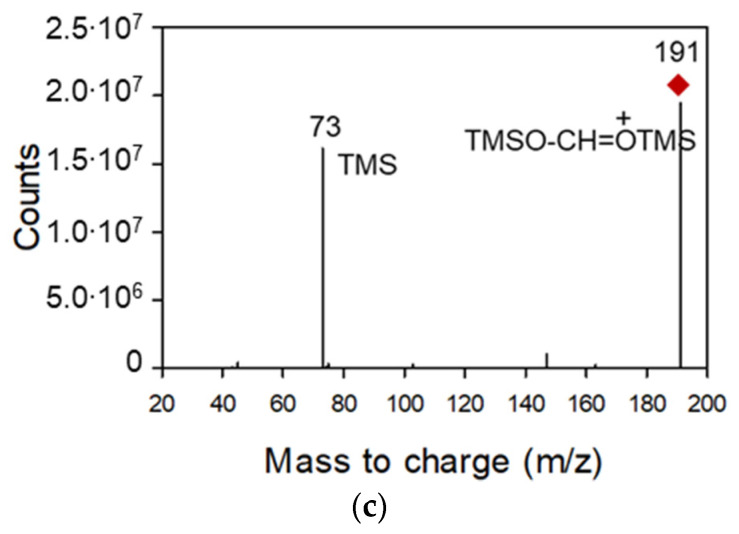
Product ion spectra of derivatized (**a**) arabinose (precursor ion: *m/z* 217), (**b**) levoglucosan (precursor ion: *m*/*z* 204), and (**c**) xylose (precursor ion: *m*/*z* 191).

Silylation-derivatized saccharides and their fragmentation patterns have been analyzed using GC-MS [22,23] and GC-MS/MS [24] in previous studies. Compared to the analytical results of saccharides in food samples using GC-MS/MS [24], the detection limits were lower in this study, as shown in Appendix B. However, the saccharides in ambient particulate matter have rarely been analyzed using GC-MS/MS. In this study, the precursor ions (the common peaks of derivatized saccharides, demonstrated in previous studies using GC-MS) [9,22] were designated as follows: arabinose, levoglucosan, and sucrose (*m*/*z* 217); arabinose, ribose, xylose, fructose, galactose, and maltose (*m*/*z* 204); and xylose, mannose, and glucose (*m*/*z* 191). Trimethylsilyl (TMS) (*m*/*z* 73) was selected as a product ion, which was the most abundant among the detected product ions, as shown in Figure 2. 

In MRM mode, the precursor ions of the target saccharides were selected in the first quadrupole (Q1) and dissociated in the second quadrupole (Q2), and the product ions from each saccharide were identified and quantified in the third quadrupole (Q3). By specifying certain precursor ions and specific masses, this data acquisition mode results in increased sensitivity and structural specificity for the analyte [25]. Even though the precursor and product ions were common fragment ions in the analysis of silylation-derivatized saccharides in atmospheric aerosols, the selectivity and sensitivity in MRM mode resulted in more distinct target peaks that were less affected by interference than those in SIM mode, as the baselines of GC-MS/MS in MRM mode were lower. 

**Figure 3:**
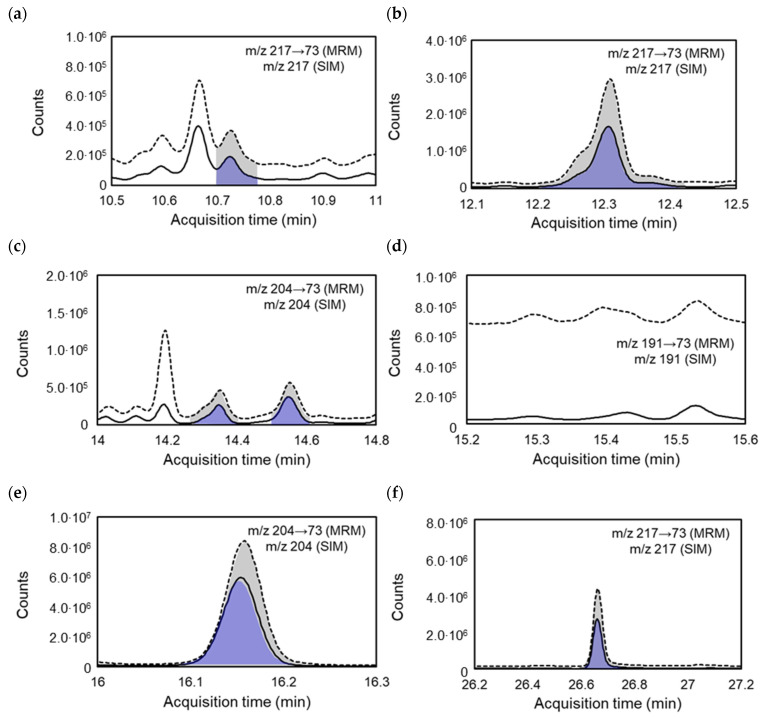

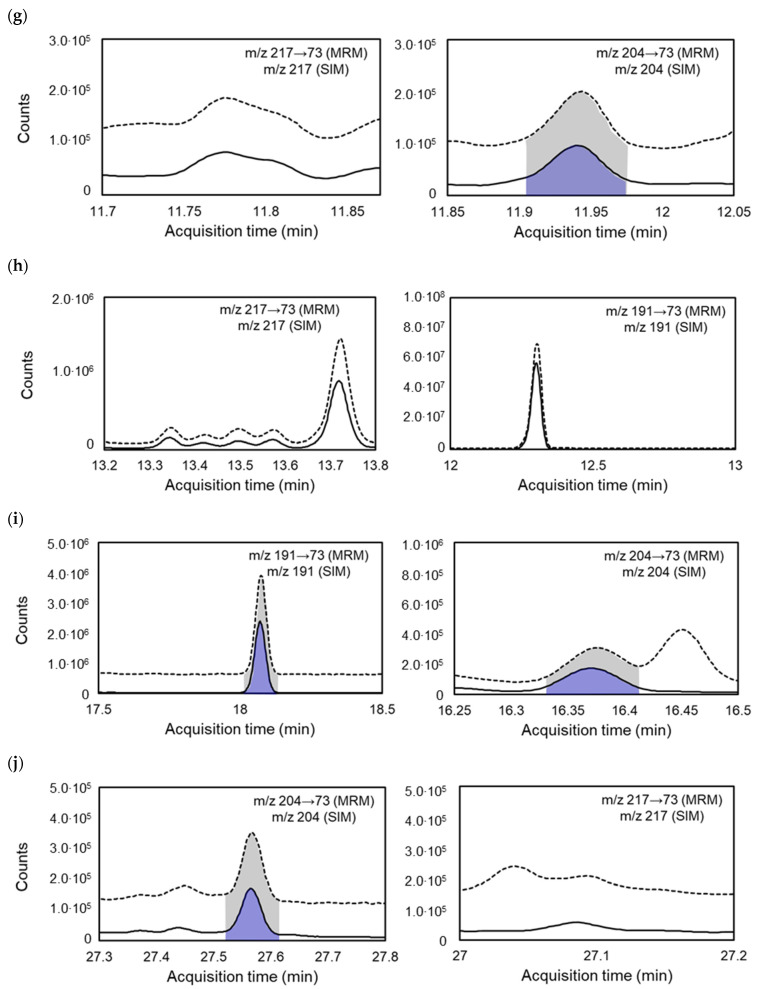
Chromatographic separations obtained using GC-MS/MS in MRM mode (solid line) and GC-MS in SIM mode (dotted line) for (**a**) arabinose, (**b**) levoglucosan, (**c**) fructose, (**d**) mannose, (**e**) galactose, (**f**) sucrose, (**g**) ribose, (**h**) xylose, (**i**) glucose, and (**j**) maltose in sample #11.

Levoglucosan, fructose, and mannose in sample #11, which contained the lowest OC content among the collected samples, could not be quantified or identified using GC-MS. However, these compounds were quantifiable when using GC-MS/MS. As shown in Figure 3, the baselines were lowered by using two parallel mass filters for GC-MS/MS, and as a result, the signal-to-noise (S/N) ratios were generally increased, except for a peak of galactose (retention time: 16.2 min) (Table 7).

### 3.2. Enhanced Quantification and Identification Using GC-MS/MS 

As discussed in Section 3.1, due to the lower baseline, the MDLs of GC-MS/MS for the target saccharides were lower than those of GC-MS. The MDLs obtained using GC-MS/MS in MRM mode ranged from 1.38 pg/μL (mannose) to 11.7 pg/μL (sucrose), whereas those measured using GC-MS in SIM mode ranged from 5.57 pg/μL (mannose) to 31.37 pg/μL (sucrose). Thus, the MDLs of GC-MS/MS in MRM mode were between 2 (galactose) and 22 (mannose) times lower than those of GC-MS in SIM mode, as reported in Table 5. Because the MDLs of GC-MS were higher than those of GC-MS/MS, the minimum values in the linear ranges of GC-MS were generally higher than those of GC-MS/MS for target saccharides such as ribose, levoglucosan, xylose, mannose, glucose, and sucrose. 

The MDLs measured in this study using the two techniques were compared to those obtained with HPAEC-MS, which was used in a previous study to analyze saccharides in PM samples collected in the Antarctic region [10]. -. The MDLs measured in this study using GC-MS/MS in MRM mode were lower than those reported by Barboro et al. (2015) for HPAEC-MS [10]. For example, levoglucosan, which is an important biomass burning marker, had a GC-MS/MS MDL of 1 ng, which was one-fifth (1/5) of the value obtained with GC-MS (5 ng). In addition, the MDLs of GC-MS/MS for galactose, glucose, and sucrose were lowered by factors of 10–30 relative to GC-MS values. The MDLs for arabinose, ribose, levoglucosan, xylose, and fructose were similar between GC-MS in SIM mode and HPAEC-MS (reported by Barboro et al., 2015) [10], but the MDLs of GC-MS in SIM mode were lower for galactose, glucose, and sucrose and higher for mannose compared to those of HPAEC-MS. 

These results indicate that GC-MS/MS is more suitable than GC-MS or HPAEC-MS for the analysis of saccharides in samples collected in marine and remote regions (which might contain only trace amounts of saccharides). The MDL concentration ranges of the target saccharides are similar between GC-MS and HPAEC-MS, so the technique should be selected according to the target saccharides, as well as the preferred type of chromatographic technique and sample preparation steps. 

The mean of the sum of the target saccharides quantified using GC-MS/MS in MRM mode was 2778.2 ± 4946.9 pg/m^3^ (Table 8). Sample #8, which was collected near the seashore of New Zealand, had the highest sum of target saccharide concentrations (16,569.9 pg/m^3^), and sample #12, collected in the South Pacific Ocean, had the lowest (159.8 pg/m^3^). Among the saccharides, the mean concentration of glucose was the highest (935.6 ± 1646.5 pg/m^3^), and that of fructose was the lowest (29.9 ± 44.8 pg/m^3^). The average concentrations of the sum of the saccharide concentrations measured using GC-MS in SIM mode (2600.2 ± 4723.5 pg/m^3^) were lower than those measured using GC-MS/MS in MRM mode (2778.2 ± 4946.9 pg/m^3^) (Table 8). The concentrations measured using GC-MS/MS in MRM mode were 1.1-fold higher than those measured using GC-MS in SIM mode. 

The relative differences between the measured concentrations of saccharides between the two tested modes are also reported in Table 8. The saccharide concentrations of samples #1 and #8, which had high OC concentrations (measured simultaneously with saccharides), were overestimated by as much as 3–65% using GC-MS in SIM mode compared to the values measured by GC-MS/MS in MRM mode. The concentrations of glucose and sucrose in samples #11 and #12, which had low OC concentrations, were 14–51% higher when using GC-MS/MS in MRM mode compared to their values with GC-MS. The selectivity and sensitivity in MRM mode resulted in more distinct target peaks that were less affected by interference than those in SIM mode. 

Maltose was the most overestimated compound in all samples when using GC-MS in SIM mode. On the other hand, levoglucosan and sucrose exhibited higher concentrations when using GC-MS/MS in MRM mode. Differences in the concentrations of maltose, levoglucosan, and sucrose concentrations between GC-MS/MS and GC-MS decreased as the quantified concentrations increased. This indicates that the uncertainty of the quantification using GC-MS and GC-MS/MS decreases when the quantified concentrations are high. 

Galactose, glucose, sucrose, and maltose were generally detectable using either GC-MS/MS or GC-MS. Ribose was not quantifiable in samples #1, #5, and #7–12, whereas it was measured in the rest of the samples. Levoglucosan, a unique marker used to evaluate the contribution of anthropogenic emissions to pristine marine aerosols, was detected in all samples except samples #3 and #4, collected near the Philippine Sea, and samples #9, #11, and #12, collected in the Antarctic region. 

Xylose was quantifiable using GC-MS/MS in MRM mode for all samples except #8–11, which were collected near the Antarctic region, whereas GC-MS could only quantify xylose in samples collected near land (#1–2 and #5–6). Furthermore, fructose and mannose were quantifiable in all samples using GC-MS/MS, whereas GC-MS could only quantify fructose and mannose in several samples. The frequency of below detection limit (BDL) concentrations was reduced from 16% with GC-MS in SIM mode to 4% with GC-MS/MS in MRM mode. Furthermore, the frequency of concentrations that were not detected (ND) (15%) was improved when using GC-MS/MS in MRM mode (7%). 

Because the differences between the quantification results of GC-MS and GC-MS/MS were generally within ±20%, GC-MS can be considered suitable when analyzing samples with sufficiently high levels of saccharides, such as samples #1, #2, #5, #6, and #8, which were collected near land and contained large amounts of organic compounds. However, GC-MS/MS should be considered for the analysis of saccharides in PM samples collected in remote and marine environments, such as #3–6 and #9–12, where the amounts of OC are low (Table 6). 

### 3.3. Estimation of Improved Time Resolution of Sampling by Applying GC-MS/MS 

The time resolution for sampling saccharides can be improved by using GC-MS/MS. A sampling interval of 1–3 days was used for the analysis of the target saccharides because their concentrations were assumed to be too low for GC-MS. A higher time resolution can be obtained using GC-MS/MS because the MDLs are generally lower than those of GC-MS, as discussed above. 

Table 9 shows the suggested sampling times (min) required to obtain a PM_2.5_ sample using a high-volume air sampler at a flow rate of 700 L/min to enable the quantification of each saccharide using GC-MS/MS. In this study, the minimum sampling times required for the quantification of the target saccharides using GC-MS/MS and GC-MS were estimated based on their MDLs and the concentrations measured with each technique, as detailed in Table 9 (minimum sampling volume (m^3^) = MDL (pg/μL) × final extraction volume (500 μL)/measured concentration (pg/m^3^)). The flow rate of the PM_2.5_ high-volume air sampler was assumed to be 700 L/min (Sibata Scientific Technology). The empty cells in Table 9 indicate “not calculated” because the concentrations could not be quantified. 

The saccharides (except for those not quantified using GC-MS/MS or GC-MS) can be quantified in samples collected daily at sampling points #1–8. However, ribose in sample #9 and fructose in sample #12 cannot be quantified in samples collected with a daily interval because the ambient concentrations of ribose and fructose in the Antarctic region are likely to be lower than those of other saccharides ([10]; Table 1). In contrast, although the concentrations of mannose in marine and Antarctic environments were also low, mannose was detectable using GC-MS/MS because its MDL was lower than those of ribose and fructose. 

Based on the results, the time resolution of the concentrations of the target saccharides in remote and Antarctic regions can be improved from several minutes to one day, except for those of ribose and fructose, when using GC-MS/MS; the concentration of ribose in sample #9 and that of fructose in samples #11 and #12 were close to GC-MS/MS MDLs and greater than those of other saccharides. Saccharides that were not detectable using GC-MS/MS might not exist in marine and Antarctic regions. Because some of the target saccharides in the samples collected in remote and marine regions were not quantifiable using GC-MS/MS, the target saccharides selected for research require daily profiles to identify their main contributors in marine and remote regions, including Antarctica. 

## 4. Conclusions

To characterize the chemical properties of marine aerosols that can affect the global climate, a total of 10 saccharides were analyzed from 11 PM_2.5_ samples (with the exclusion of sample #10), which were collected while aboard the IBRV Araon as it sailed from Incheon, Korea, to the Antarctic Jang-Bogo Station (from 31 October to 14 December 2018). The identification and quantification of 10 saccharide concentrations using GC-MS/MS in MRM mode and GC-MS in SIM mode were compared and evaluated. The results indicate that GC-MS/MS is an appropriate technique for the analysis of samples with trace saccharides in the Antarctic marine environment.

An advantage of analyzing saccharides using GC-MS/MS in MRM mode instead of GC-MS is the lower baseline of the chromatogram, which enables the accurate identification of these analytes. Specifically, the peaks of levoglucosan in sample #11, the first peak of fructose in samples #8 and #11, and the second peak of glucose in sample #11 were not contaminated by interference when using GC-MS/MS, leading to the accurate quantification of these saccharides. In contrast, these peaks were affected by interference in GC-MS chromatograms.

The lower baselines in the chromatograms obtained with GC-MS/MS in MRM mode decreased the MDLs of the target saccharides by as much as 2–22-fold compared to those of GC-MS in SIM mode. The lower MDLs enabled the quantification of the target saccharides, the concentrations of which were below the GC-MS MDLs in SIM mode. The frequency of BDL values was reduced from 16% when using GC-MS in SIM mode to 4% when using GC-MS/MS in MRM mode. Furthermore, the frequency of ND values (15%) was improved when using GC-MS/MS in MRM mode (7%). Differences in the concentrations of saccharides such as levoglucosan, glucose, and sucrose between GC-MS/MS and GC-MS decreased as the quantified concentration increased. However, differences between the concentrations of saccharides quantified using GC-MS and GC-MS/MS were within ±20%, and GC-MS in SIM mode can sufficiently quantify saccharides with levels over its corresponding MDLs. Nonetheless, GC-MS/MS is an appropriate technique for the analysis of saccharides when the amount of the organic compounds in PM is high enough to use GC-MS. 

Furthermore, the lower MDLs obtained with GC-MS/MS in MRM mode can improve the time resolution of the sampling from several minutes to one day, allowing the quantification of saccharides in samples collected in Antarctic marine regions (except ribose and fructose). Due to the lower chromatographic baseline and, consequently, its lower MDLs, GC-MS/MS can provide the daily profiles of saccharide concentrations in PM samples collected in the Antarctic region. However, the target saccharides, the analytical technique, and preferred sample preparation steps should be selected according to the research goals. 

The concentrations of saccharides in Antarctica that are quantifiable with GC-MS/MS can be used to indicate the emission sources and characterize the atmospheric reactions that form aerosols in pristine marine environments. Furthermore, studying the main contributors of saccharides can help clarify the impact of aerosols on climate change. Future studies that identify contributors of aerosols containing saccharides should examine potential associations with the observed behaviors of microorganisms and other compounds collected in the marine environment.

## Figures and Tables

**Table 1:** The analytical method, method detection limit, recovery, and ambient concentration of saccharides in particulate matter samples collected in polar environments and urban areas in previous studies.

Reference	Nolte et al., 2001	Wan and Yu, 2006	Mederios and Simoneit, 2007	Yttri et al., 2007	Fu et al., 2009	Wang et al., 2011	Barbaro et al., 2015	Choi et al., 2016
The number of target sugars	8	9	13	7	8	11	8	6
Sample type	Fine particulate matter with an aerodynamic diameter less than 2.0 μm	PM_2.5_^1^	Bulk aerosol (>1 μm)	PM_10_^2^, PM_2.5_	TSP ^3^	Size segmented mode (cutoff points of 0.4, 0.7, 1.1, 2.1, 3.3, 4.7, 5.8, and 9.0 μm)	TSP ^3^	PM_10_^2^
Sampling site	California (urban)	Hong Kong (urban)	Howland Experimental Forest, Maine, USA	Oslo, Norway (urban)	The France–Canada–USA joint Arctic campaign, Canadian Artic	NanJing (urban)	Mario Zucchelli Station, Antarctica	Seoul, South Korea (urban)
Sampling period	1995.12–1996.01	2004.09–2005.04	2002.05–2002.08	2002.09–2002.10.	Summer in 2009	-	2010.11.29–2012.01.28	2010.04–2011.04
Pretreatment	Solvent extraction with derivatization using BSTFA ^4^ (70 °C, 2 h) and 1% TMCS ^5^	Solvent extraction	Solvent extraction with derivatization using BSTFA ^4^ (70 °C, 3 h) and 1% TMCS ^5^	Solvent extraction	Solvent extraction with the derivatization using N, O-bistrifluoroacetamide (BSTFA ^4^; 70 °C, 3 h) and 1% trimethylchlorosilane (TMCS) ^5^	Solvent extraction with derivatization using BSTFA ^4^ (70 °C, 3 h) and 1% TMCS ^5^	Solvent extraction	Solvent extraction with derivatization using BSTFA ^4^ (75 °C, 1 h) and 1% TMCS ^5^
Instrument	GC-MS ^6^	LC-MS ^7^	GC-MS ^6^	HPLC-HRMS/TOF ^8^	GC-MS ^6^	GC-MS ^6^	HPAEC-MS ^9^	GC-MS ^6^
MDL	-	0.014–0.95 pmol/uL	130–280 pg/m^3^	30 pg	-	-	3–60 ng	0.04–0.186 ng/uL
Recovery	-	94–112%	-	-	>80%	>80%	-	64–113%
Concentration	ND ^10^-2980 ng/m^3^	39–1310 ng/m^3^	ND ^10^-55.1 ng/m^3^	ND ^10^-7.2	Not detected (ND ^10^)-8.6 ng/m^3^	16–4030 ng/m^3^ (sum of compound in the 9 size-resolved stages; haze, summer)	ND ^10^-664.4 ng/m^3^	5.06–387.49 ng/m^3^

^1^ Particulate matter with an aerodynamic diameter equal or less than 2.5 μm; ^2^ Particulate matter with an aerodynamic diameter equal or less than 10 μm; ^3^ Total suspended particles; ^4^ BSTFA; ^5^ TMCS; ^6^ Gas chromatography-mass spectrometry; ^7^ Liquid chromatography-mass spectrometry; ^8^ High-performance liquid chromatography-high-resolution mass spectrometry/time-of-flight mass spectrometry; ^9^ High-performance anion-exchange chromatography-mass spectrometry; ^10^ Not detected.

**Table 2:** The chemical properties and structures of 10 target saccharides in this study.

Compounds	Abbreviation	Formula	CAS Number	Molecular Weight (g/mol)	Boiling Point (°C; at 760 mmHg)	Water Solubility (mol/L; at 25 °C)	Structures
D-(-)-Arabinose	Arabinose	C_5_H_10_O_5_	10323-20-3	150.1299	317	3.49	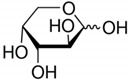
D-(-)-Ribose	Ribose	C_5_H_10_O_5_	50-69-1	150.1299	341	9.51	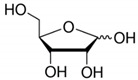
Levoglucosan	-	C_6_H_10_O_5_	498-07-7	162.1406	308	0.68	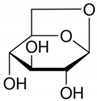
D-(+)-Xylose	Xylose	C_5_H_10_O_5_	58-86-6	150.1299	309	1.96	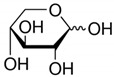
D-(-)-Fructose	Fructose	C_6_H_12_O_6_	57-48-7	180.1559	355	1.59	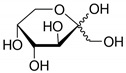
D-(+)-Mannose	Mannose	C_6_H_12_O_6_	3458-28-4	180.1559	348	2.93	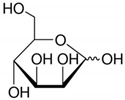
D-(+)-Galactose	Galactose	C_6_H_12_O_6_	59-23-4	180.1559	348	2.93	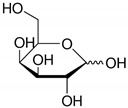
D-(+)-Glucose	Glucose	C_6_H_12_O_6_	50-99-7	180.1559	384	3.30	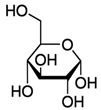
Sucrose	-	C_12_H_22_O_11_	57-50-1	342.2965	472	2.70	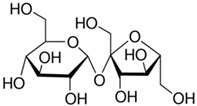
D-(+)-Maltose	Maltose	C_12_H_22_O_11_	69-79-4	342.2965	467	1.34	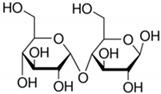

**Table 3:** Sampling information, including sampling time, location, volume, and route.

Sample Number	Start UTC ^a^ (Location)	Finish UTC (Location)	Sampling Volume (m^3^)	Route	Note
1	2018-10-31 6:00 (37.21° N, 126.31° E)	2018-11-02 0:00 (29.15° N, 129.35° E)	-	Yellow Sea– East China Sea	The flow rate could not be measured.
2	2018-11-02 1:16 (29.15° N, 129.35° E)	2018-11-03 21:53 (21.37° N, 134.55° E)	2541.1	East China Sea–Philippine Sea	-
3	2018-11-03 22:19 (21.37° N, 134.55° E)	2018-11-06 2:10 (12.54° N, 141.09° E)	2989.3	Philippine Sea	-
4	2018-11-06 2:37 (12.54° N, 141.09° E)	2018-11-08 5:30 (4.15° N. 147.30° E)	2740.0	Philippine Sea–Pacific Ocean	-
5	2018-11-08 6:00 (4.15° N, 147.30° E)	2018-11-12 0:04 (12.31° S, 157.22° E)	3070.2	North Pacific Ocean– Coral Sea	-
6	2018-11-12 0:25 (12.31° S, 157.22° E)	2018-11-15 2:37 (24.46° S, 165.06° E)	3672.5	Coral Sea– Coral Sea	-
7	2018-11-15 3:05 (24.46° S, 165.06° E)	2018-11-19 1:15 (42.41° S, 173.36° E)	1132.0	Coral Sea–Seashore of New Zealand ^b^	-
8	2018-11-19 1:37 (42.41° S, 173.36° E)	2018-11-25 2:42 (44.00° S, 173.10° E)	2246.4	Seashore of New Zealand	-
9	2018-11-25 3:06 (44.00° S, 173.10° E)	2018-12-01 3:51 (74.41° S, 164.10° E)	3099.7	Seashore of New Zealand–Antarctica ^c^	-
10	2018-12-02 21:00 (74.41° S, 164.10° E)	2018-12-07 23:45 (74.50° S, 165.01° E)	-	Antarctica (Anchorage)	Filter was damaged
11	2018-12-08 0:02 (S74.50° S, 165.01° E)	2018-12-11 8:30 (61.25° S, 177.35° E)	2588.8	Antarctica–South Pacific Ocean	-
12	2018-12-11 8:45 (48.04° S, 177.35° E)	2018-12-14 8:52 (48.04° S, 178.26° E)	3001.1	South Pacific Ocean	-

^a^ Universal Coordinated Time; ^b^ Northeastern seashore of New Zealand’s South; ^c^ Jang Bogo Station, Antarctica.

**Table 4:** Selected ion monitoring (SIM) conditions used in MS analysis and multiple reaction monitoring (MRM) conditions used in MS/MS analysis of target saccharides.

Compound	Retention Time (min)	SIM	MRM		
		Selected Ion (*m/z*)	Precursor Ion (*m/z*)	Product Ion (*m/z*)	Collision Energy (eV)
Arabinose *	10.7	191, 204, 217	217	73	10
	10.9	191, 204, 217	204	73	10
Ribose *	11.7	191, 204, 217	204	73	10
	11.9	191, 204, 217	204	73	10
Levoglucosan	12.3	204, 217, 333	217	73	10
Xylose *	12.5	191, 204, 217	191	73	10
	13.5	191, 204, 217	204	73	10
Fructose *	14.4	147, 204, 437	204	73	5
	14.5	147, 204, 437	204	73	5
Mannose	15.4	191, 204, 217	191	73	10
Galactose	16.2	191, 204, 217	204	73	5
Glucose *	16.4	191, 204, 217	204	73	5
	18.1	191, 204, 217	191	73	5
Sucrose *	26.6	147, 217, 361	217	73	10
Maltose *	27.1	147, 217, 361	217	73	10
	27.6	191, 204, 217	204	73	10

* Both α- and β-isomers were assumed.

**Table 5:** MDLs (*n* = 10) and linear ranges of MS (at a level of 30 pg/m^3^) and MS/MS (at a level of 6 pg/m^3^) for the analysis of saccharides in this study and MDLs measured using HPAEC-MS in Barboro et al., 2015 [10].

Compound	GC-MS/MS			GC-MS		
	This Study			This Study		
	MDL (pg/uL)	MDL (ng) *	Linear Range (pg/uL)	MDL (pg/uL)	MDL (ng) *	Linear Range (pg/uL)
Arabinose	2.21	1	6–2500	8.28	4	60–1250
Ribose	2.04	1	6–1250	9.36	5	60–1250
Levoglucosan	2.10	1	6–1250	15.78	8	60–1250
Xylose	3.29	2	6–5000	9.45	5	60–5000
Fructose	2.55	1	6–1250	5.57	3	60–1250
Mannose	1.38	1	6–5000	31.37	16	60–1250
Galactose	5.99	3	6–5000	11.44	6	60–1500
Glucose	2.38	1	6–5000	7.76	4	60–5000
Sucrose	11.70	6	6–5000	23.44	12	60–5000
Maltose	1.99	1	6–1250	11.90	6	60–1250

* Recalculated and multiplied by a final extraction volume of 500 μL.

**Table 6:** Organic carbon (OC) and elemental carbon (EC) concentrations measured in samples collected during the Araon cruise in 2018 (unit: μg/m^3^).

Sample Number	OC	EC
#2	0.49	0.03
#3	0.13	0.01
#4	0.10	BDL *
#5	0.11	0.01
#6	0.07	0.01
#7	0.15	BDL
#8	1.33	0.17
#9	0.05	BDL
#11	0.04	BDL
#12	0.05	BDL

* Below detection limit.

**Table 7:** Signal-to-noise ratios of target saccharides in sample #11 when using GC-MS/MS and GC-MS.

Compound	Retention Time (min)	GC-MS/MS (MRM Mode)	GC-MS (SIM Mode)
Arabinose	10.7	5.1	0.1
	10.9	5.7	0.1
Ribose	11.7	1.4	- *
	11.9	0.4	-
Levoglucosan	12.3	51.1	16.1
Xylose	12.5	0.3	0
	13.5	1.7	0
Fructose	14.4	1.4	0.2
	14.5	1.9	0.2
Mannose	15.4	0.4	-
Galactose	16.2	33.7	62.1
Glucose	16.4	0.8	0.1
	18.1	11.1	10.9
Sucrose	26.6	83.1	24.2
Maltose	27.1	0.9	-
	27.6	2.7	0.1

* Not calulated.

**Table 8:** Concentrations of target saccharides in samples collected on the Araon using GC-MS/MS in MRM mode and GC-MS in SIM mode (unit: pg/m^3^) and the differences in concentration between the two modes. Samples were collected while sailing from Incheon, South Korea, to the Antarctic region.

Compound		#2	#3	#4	#5	#6	#7	#8	#9	#11	#12	Average	Stdev ^a^
Arabinose	MRM	177.0	3.7	BDL ^b^	167.0	341.4	BDL	25.8	BDL	BDL	BDL	143.0	136.2
	SIM	184.5	4.4	ND ^c^	135.1	291.2	BDL	26.7	BDL	ND	BDL	128.4	117.7
	MRM/ SIM	1.0	1.2	-	0.8	0.9	-	1.0	-	-	-	0.9	-
Ribose	MRM	ND	ND	ND	8.5	ND	6.3	1097.9	1.7	BDL	BDL	278.6	546.2
	SIM	ND	ND	ND	BDL	ND	BDL	1063.1	BDL	ND	BDL	1063.1	-
	MRM/ SIM	-	-	-	-	-	-	-	-	-	-	3.8	-
Levoglucosan	MRM	372.7	5.0	3.2	84.4	24.4	55.2	2174.1	3.8	19.4	4.5	274.7	676.8
	SIM	342.8	BDL	BDL	50.2	22.2	34.2	2234.4	BDL	BDL	BDL	536.9	958.3
	MRM/ SIM	0.9	-	-	0.6	0.9	0.6	1.0	-	-	-	2.0	-
Xylose	MRM	139.2	9.7	5.8	144.2	179.7	9.5	ND	ND	BDL	BDL	81.4	81.2
	SIM	106.5	ND	BDL	127.1	190.8	BDL	ND	ND	ND	ND	141.5	44.0
	MRM/ SIM	0.8	-	-	0.9	1.1	-	-	-	-	-	1.7	-
Fructose	MRM	33.9	1.7	4.2	92.1	19.1	9.03	129.8	4.7	3.1	1.6	29.9	44.8
	SIM	31.5	BDL	5.6	77.5	23.5	11.5	136.3	5.1	BDL	BDL	41.6	48.7
	MRM/ SIM	0.9	-	1.3	0.8	1.2	1.3	1.1	1.1	-	-	1.4	-
Mannose	MRM	216.29	1.11	3.98	58.70	34.69	15.12	122.87	3.43	3.29	5.54	46.50	70.87
	SIM	217.21	ND	ND	56.93	42.22	ND	142.12	ND	ND	ND	114.62	81.34
	MRM/ SIM	1.0	-	-	1.0	1.2	-	1.7	-	-	-	2.5	-
Galactose	MRM	358.4	35.6	137.9	758.3	501.8	582.3	3931.9	157.2	130.8	31.4	662.6	1175.1
	SIM	356.3	35.2	154.8	748.7	562.6	693.2	4101.3	165.3	131.2	34.1	698.3	1224.8
	MRM/ SIM	1.0	1.0	1.1	1.0	1.1	1.2	1.0	1.1	1.0	1.1	1.1	-
Glucose	MRM	356.8	66.7	97.6	758.4	1743.5	576.5	5384.4	243.2	104.0	25.3	935.6	1646.5
	SIM	285.0	49.8	76.1	500.7	1504.0	467.0	4370.3	199.4	74.3	12.5	753.9	1345.0
	MRM/ SIM	1.0	0.8	0.8	0.7	0.9	0.8	0.8	0.8	0.7	0.5	0.8	-
Sucrose	MRM	91.0	132.5	144.3	156.4	19.1	1022.5	3559.4	198.6	98.7	87.5	551.0	1095.8
	SIM	80.7	95.9	126.2	135.1	14.3	998.2	3523.7	191.1	84.6	73.6	532.4	1089.3
	MRM/ SIM	0.9	0.8	0.9	0.9	0.8	1.0	1.0	1.0	0.9	0.8	1.0	-
Maltose	MRM	68.5	43.8	107.9	59.0	ND	15.2	143.8	13.3	6.5	3.4	51.3	49.0
	SIM	112.6	51.5	131.2	67.0	ND	19.5	186.6	13.9	7.4	3.7	65.9	64.6
	MRM/ SIM	1.7	1.2	1.2	1.1	-	1.3	1.3	1.1	1.1	0.9	1.3	-
Sum	MRM	1813.6	299.8	505.0	2287.0	2863.7	2291.7	16,569.9	625.9	365.7	159.8	2778.2	4946.9
	SIM	1717.1	236.9	493.9	1898.5	2651.5	2223.6	15,784.6	574.8	297.4	123.9	2600.2	4723.5
	MRM/ SIM	1.0	0.8	1.0	0.8	0.9	1.0	1.0	0.9	0.8	0.8	0.9	-

^a^ Standard deviation; ^b^ below detection limit; ^c^ not detected.

**Table 9:** Sampling time (min) required to obtain a PM_2.5_ sample using a high-volume air sampler at a flow rate of 700 L/min to allow the quantification of saccharides using GC-MS/MS and MS.

		#2	#3	#4	#5	#6	#7	#8	#9	#11	#12
Arabinose	MS/MS	8.9	425.7	- ^*^	9.4	4.6	-	61.1	-	-	-
	MS	32.1	1349.7	-	43.8	20.3	-	221.5	-	-	-
Ribose	MS/MS	-	-	-	171.0	-	231.5	1.3	864.9	-	-
	MS	-	-	-	-	-	-	6.3	-	-	-
Levoglucosan	MS/MS	4.0	298.6	471.9	17.7	61.4	27.1	0.7	390.3	77.3	330.6
	MS	32.9	-	-	224.7	491.9	329.3	5.0	-	-	-
Xylose	MS/MS	16.9	241.3	402.4	16.3	13.1	246.4	-	-	-	-
	MS	63.4	-	-	53.1	35.4	-	-	-	-	-
Fructose	MS/MS	53.9	-	430.1	19.8	95.4	202.0	14.1	392.0	581.5	1112.9
	MS	126.1	-	714.6	51.3	169.3	345.0	29.2	782.7	-	-
Mannose	MS/MS	4.6	-	248.3	16.9	28.5	65.4	8.1	288.8	300.7	178.7
	MS	103.2	-	-	393.6	530.7	-	157.7	-	-	-
Galactose	MS/MS	11.9	120.1	31.0	5.6	8.5	7.3	1.1	27.2	32.7	136.1
	MS	22.9	231.8	52.8	10.9	14.5	11.8	2.0	49.4	62.3	239.3
Glucose	MS/MS	4.8	25.5	17.4	2.2	1.0	3.0	0.3	7.0	16.4	67.3
	MS	19.5	111.4	72.9	11.1	3.7	11.9	1.3	27.8	74.6	444.5
Sucrose	MS/MS	91.9	63.1	57.9	53.5	438.6	8.2	2.3	42.1	84.7	95.6
	MS	207.5	174.6	132.6	123.9	1169.9	16.8	4.8	87.6	198.0	227.5
Maltose	MS/MS	20.8	32.5	13.2	24.1	-	93.5	9.9	107.2	219.0	361.4
	MS	75.4	164.9	64.8	126.8	-	436.6	45.5	610.7	1155.9	2311.6

* Not calculated since the concentration was not quantified.

## Data Availability

The data presented in this study are available on request from the corresponding author. The data are not publicly available due to confidentiality.

## Appendix A

**Table A1:** MDLs measured using MS and the concentrations of saccharides in PM_10_ in Antarctic regions measured in previous studies.

Compound	MDL (pg/m^3^)			Concentration (ng/m^3^)		
				Barboro et al., 2015 ^c^ (Mario Zucchelli Station, 74.42° S, 164.06° E)		
	Choi et al., 2016 ^a^	Medeiros and Simoneit, 2007 ^b^	Barbaro et al., 2015	2010.11.29–2011.12.09	2011.12.09–2011.01.18	2011.12.19–2012.01.28
Arabinose	123	-	0.2	27.1	0.9	-
Ribose	132	-	0.3	-	-	-
Levoglucosan	122	130	0.3	13.4	0.4	-
Xylose	77	-	0.4	13.6	-	-
Fructose	186	-	0.2	149.2	0.9	11.3
Mannose	41	-	0.4	27.1	-	-
Galactose	58	-	2.0	13.6	-	-
Glucose	73	150	2.0	664.4	51.6	312.1
Sucrose	101	280	4.0	-	-	5.2
Maltose	128	-	-	-	-	-

^a^ Method detection limit; ^b^ Limit of Detection; ^c^ PM_10_ samples (sampling volume: 15,000 m^3^).

## Appendix B

**Table A2:** Detection limits of saccharides using GC-MS/MS coupled with silylation derivatization in a previous study and this study.

	Limit of Detection (pg/μL)	Method Detection Limit (pg/μL)
	Gómez-González et al., 2010	This Study
Arabinose	76	2.21
Xylose	76	3.29
Glucose	15	2.38
Mannose	30	1.38
Galactose	76	5.99
Fructose	30	2.55
Sucrose	15	11.70
